# Tetra-μ-acetato-κ^8^ *O*:*O*′-bis[(*N* ^2^,*N* ^2^-di­methyl­pyrazin-2-amine-κ*N* ^4^)copper(II)]

**DOI:** 10.1107/S160053680901719X

**Published:** 2009-05-14

**Authors:** Lin Meng, Lin Yan Yang, Jing Min Shi

**Affiliations:** a Department of Chemistry, Shandong Normal University, Jinan 250014, People’s Republic of China

## Abstract

The title binuclear complex, [Cu_2_(C_2_H_3_O_2_)_4_(C_6_H_9_N_3_)_2_], lies on an inversion center with four acetate ligands bridging two Cu^II^ ions and two monodentate *N*,*N*-dimethyl­pyrazine-2-amine ligands coordinating each Cu^II^ ion *via* N atoms, forming slightly distorted square-pyramidal environments.

## Related literature

For related structures, see: Zhang *et al.* (2007 ▶); Li *et al.* (2003 ▶).
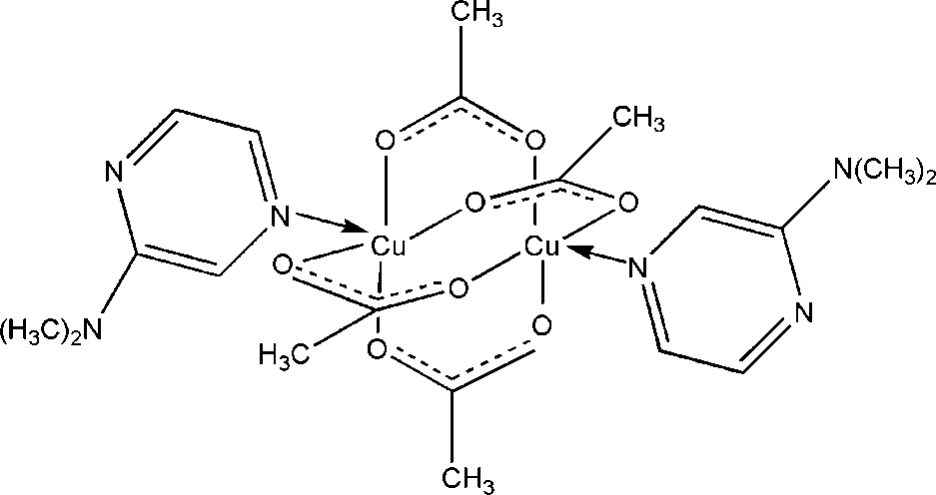


## Experimental

### 

#### Crystal data

[Cu_2_(C_2_H_3_O_2_)_4_(C_6_H_9_N_3_)_2_]
*M* *_r_* = 609.58
Triclinic, 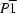

*a* = 8.1052 (13) Å
*b* = 8.1775 (13) Å
*c* = 10.6534 (17) Å
α = 67.826 (2)°
β = 80.013 (2)°
γ = 87.328 (2)°
*V* = 643.84 (18) Å^3^
*Z* = 1
Mo *K*α radiation
μ = 1.71 mm^−1^
*T* = 298 K
0.68 × 0.41 × 0.31 mm

#### Data collection

Bruker SMART APEX CCD diffractometer
Absorption correction: multi-scan (*SADABS*; Sheldrick, 1996 ▶) *T*_min_ = 0.390, *T*_max_ = 0.620
3494 measured reflections
2465 independent reflections
2317 reflections with *I* > 2σ(*I*)
*R*_int_ = 0.016

#### Refinement

*R*[*F* ^2^ > 2σ(*F* ^2^)] = 0.032
*wR*(*F* ^2^) = 0.094
*S* = 1.09
2465 reflections
167 parameters
H-atom parameters constrained
Δρ_max_ = 0.54 e Å^−3^
Δρ_min_ = −0.44 e Å^−3^

### 

Data collection: *SMART* (Bruker, 1997 ▶); cell refinement: *SAINT* (Bruker, 1997 ▶); data reduction: *SAINT*; program(s) used to solve structure: *SHELXTL* (Sheldrick, 2008 ▶); program(s) used to refine structure: *SHELXTL*; molecular graphics: *SHELXTL*; software used to prepare material for publication: *SHELXTL*.

## Supplementary Material

Crystal structure: contains datablocks I, global. DOI: 10.1107/S160053680901719X/lh2807sup1.cif

Structure factors: contains datablocks I. DOI: 10.1107/S160053680901719X/lh2807Isup2.hkl

Additional supplementary materials:  crystallographic information; 3D view; checkCIF report

## Figures and Tables

**Table:** 

Cu1—O3	1.9649 (18)
Cu1—O1	1.9654 (19)
Cu1—O2	1.9738 (18)
Cu1—O4	1.9756 (18)
Cu1—N1	2.197 (2)

**Table:** 

O3—Cu1—O1	168.16 (8)
O3—Cu1—O2	89.93 (9)
O1—Cu1—O2	90.44 (9)
O3—Cu1—O4	88.93 (8)
O1—Cu1—O4	88.32 (9)
O2—Cu1—O4	168.35 (7)
O3—Cu1—N1	94.74 (8)
O1—Cu1—N1	97.07 (8)
O2—Cu1—N1	92.23 (8)
O4—Cu1—N1	99.42 (8)

**Fig. 1.**
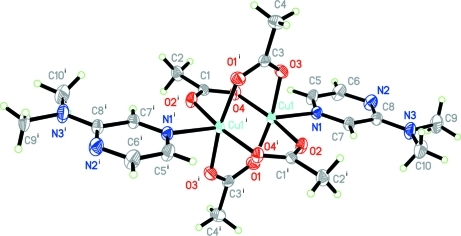
The molecular structure of title compound with the atom-numbering scheme. Displacement ellipsoids are drawn at the 30% probability level. Primed atoms are related by the symmetry operator (-x + 1, -y + 1, -z + 2).

## supplementary crystallographic information

### Comment

Both acetate anions and pyrazine derivatives are useful ligands and a large number of multi-atom complexes have been synthesized with these as bridging ligands (Zhang *et al.*, 2007; Li *et al.*, 2003). We attempted to synthesize a mixed bridged multi-nuclear Cu^II^ complex by using acetate and *N*,*N*-dimethylpyrazine-2-amine as bridging ligands. The title complex was obtained and here we report its crystal structure, (I), Fig. 1.

The unique Cu^II^ ion is in a slightly distorted square-pyramidal coordination geometry with atom N1 lying at the apex. Four acetate ligands coordinate to two symmetry-related Cu^II^ atoms, with a Cu1···Cu1^i^ separation of 2.6326 (6) Å and inversion centre lies at the middle of the Cu1···Cu1^i^ vector (symmetry code, (i): -*x* + 1, -*y* + 1, -*z* + 2) resulting in the formation of a binuclear complex. The title complex is similar to a reported binuclear Cu^II^ complex (Zhang *et al.*, 2007) except the title complex exhibits a slightly shorter Cu—N bond and a slightly longer Cu—Cu distance.

### Experimental

*N*,*N*-dimethylpyrazine-2-amine (0.0954 g, 0.0696 mmol) was dissolved in 10 ml methanol and it was added into 10 ml water solution containing copper acetate (0.1390 g, 0.696 mmol), and the mixed solution was stirred for a few minutes. The blue single crystals were obtained after the solution had been allowed to stand at room temperature for five months.

### Refinement

All H atoms were placed in calculated positions and refined as riding with C—H = 0.96 Å, *U*_iso_ = 1.5*U*_eq_(C)for methyl group and C—H = 0.93 Å, *U*_iso_ = 1.2*U*_eq_(C) for pyrazinyl H atoms.

### Crystal data

**Table:**

[Cu_2_(C_2_H_3_O_2_)_4_(C_6_H_9_N_3_)_2_]	*Z* = 1
*M**_r_* = 609.58	*F*(000) = 314
Triclinic, *P*1	*D*_x_ = 1.572 Mg m^−^^3^
Hall symbol: -P 1	Mo *K*α radiation, λ = 0.71073 Å
*a* = 8.1052 (13) Å	Cell parameters from 2730 reflections
*b* = 8.1775 (13) Å	θ = 2.7–28.2°
*c* = 10.6534 (17) Å	µ = 1.71 mm^−^^1^
α = 67.826 (2)°	*T* = 298 K
β = 80.013 (2)°	Block, blue
γ = 87.328 (2)°	0.68 × 0.41 × 0.31 mm
*V* = 643.84 (18) Å^3^	

### Data collection

**Table:**

Bruker SMART APEX CCD diffractometer	2465 independent reflections
Radiation source: fine-focus sealed tube	2317 reflections with *I* > 2σ(*I*)
graphite	*R*_int_ = 0.016
φ and ω scans	θ_max_ = 26.0°, θ_min_ = 2.1°
Absorption correction: multi-scan (*SADABS*; Sheldrick, 1996)	*h* = −9→8
*T*_min_ = 0.390, *T*_max_ = 0.620	*k* = −10→8
3494 measured reflections	*l* = −12→13

### Refinement

**Table:**

Refinement on *F*^2^	Primary atom site location: structure-invariant direct methods
Least-squares matrix: full	Secondary atom site location: difference Fourier map
*R*[*F*^2^ > 2σ(*F*^2^)] = 0.032	Hydrogen site location: inferred from neighbouring sites
*wR*(*F*^2^) = 0.094	H-atom parameters constrained
*S* = 1.09	*w* = 1/[σ^2^(*F*_o_^2^) + (0.0576*P*)^2^ + 0.297*P*] where *P* = (*F*_o_^2^ + 2*F*_c_^2^)/3
2465 reflections	(Δ/σ)_max_ = 0.020
167 parameters	Δρ_max_ = 0.54 e Å^−^^3^
0 restraints	Δρ_min_ = −0.44 e Å^−^^3^

### Special details

**Table:**

Geometry. All e.s.d.'s (except the e.s.d. in the dihedral angle between two l.s. planes) are estimated using the full covariance matrix. The cell e.s.d.'s are taken into account individually in the estimation of e.s.d.'s in distances, angles and torsion angles; correlations between e.s.d.'s in cell parameters are only used when they are defined by crystal symmetry. An approximate (isotropic) treatment of cell e.s.d.'s is used for estimating e.s.d.'s involving l.s. planes.
Refinement. Refinement of *F*^2^ against ALL reflections. The weighted *R*-factor *wR* and goodness of fit *S* are based on *F*^2^, conventional *R*-factors *R* are based on *F*, with *F* set to zero for negative *F*^2^. The threshold expression of *F*^2^ > σ(*F*^2^) is used only for calculating *R*-factors(gt) *etc*. and is not relevant to the choice of reflections for refinement. *R*-factors based on *F*^2^ are statistically about twice as large as those based on *F*, and *R*- factors based on ALL data will be even larger.

### Fractional atomic coordinates and isotropic or equivalent isotropic displacement parameters (Å^2^)

**Table:**

	*x*	*y*	*z*	*U*_iso_*/*U*_eq_	
C1	0.2373 (3)	0.6709 (3)	0.9386 (3)	0.0309 (5)	
C2	0.0727 (3)	0.7590 (4)	0.9109 (3)	0.0471 (7)	
H2A	0.0903	0.8578	0.8247	0.071*	
H2B	0.0273	0.7995	0.9832	0.071*	
H2C	−0.0043	0.6759	0.9072	0.071*	
C3	0.6075 (3)	0.7439 (3)	1.0579 (3)	0.0362 (6)	
C4	0.6713 (4)	0.8898 (4)	1.0918 (4)	0.0531 (8)	
H4A	0.7270	0.9792	1.0090	0.080*	
H4B	0.7487	0.8427	1.1548	0.080*	
H4C	0.5788	0.9406	1.1330	0.080*	
C5	0.6480 (4)	0.8180 (4)	0.5613 (3)	0.0480 (7)	
H5	0.5351	0.8437	0.5768	0.058*	
C6	0.7422 (4)	0.9016 (4)	0.4350 (3)	0.0577 (9)	
H6	0.6911	0.9856	0.3675	0.069*	
C7	0.8757 (3)	0.6637 (4)	0.6357 (3)	0.0367 (6)	
H7	0.9256	0.5812	0.7048	0.044*	
C8	0.9720 (3)	0.7479 (4)	0.5042 (3)	0.0413 (6)	
C9	1.2305 (5)	0.8021 (6)	0.3383 (4)	0.0780 (12)	
H9A	1.1700	0.7980	0.2696	0.117*	
H9B	1.3370	0.7474	0.3294	0.117*	
H9C	1.2478	0.9229	0.3261	0.117*	
C10	1.2205 (4)	0.5823 (6)	0.5770 (4)	0.0647 (9)	
H10A	1.2374	0.6312	0.6430	0.097*	
H10B	1.3270	0.5564	0.5338	0.097*	
H10C	1.1538	0.4757	0.6228	0.097*	
Cu1	0.57009 (3)	0.57928 (4)	0.87055 (3)	0.02811 (13)	
N1	0.7160 (3)	0.6997 (3)	0.6627 (2)	0.0350 (5)	
N2	0.9033 (4)	0.8693 (4)	0.4033 (3)	0.0548 (7)	
N3	1.1350 (3)	0.7085 (4)	0.4739 (3)	0.0577 (7)	
O1	0.4726 (3)	0.3816 (3)	0.8439 (2)	0.0446 (5)	
O2	0.7587 (2)	0.4311 (3)	0.94000 (19)	0.0408 (4)	
O3	0.6383 (2)	0.7576 (3)	0.9348 (2)	0.0411 (4)	
O4	0.3574 (2)	0.7044 (2)	0.83984 (19)	0.0379 (4)	

### Atomic displacement parameters (Å^2^)

**Table:**

	*U*^11^	*U*^22^	*U*^33^	*U*^12^	*U*^13^	*U*^23^
C1	0.0290 (12)	0.0292 (11)	0.0330 (13)	−0.0015 (9)	−0.0048 (10)	−0.0099 (10)
C2	0.0300 (13)	0.0478 (16)	0.0536 (18)	0.0026 (11)	−0.0087 (12)	−0.0076 (13)
C3	0.0276 (12)	0.0386 (14)	0.0479 (16)	0.0033 (10)	−0.0077 (11)	−0.0221 (12)
C4	0.0561 (18)	0.0498 (17)	0.063 (2)	−0.0056 (14)	−0.0098 (15)	−0.0309 (15)
C5	0.0421 (15)	0.0538 (17)	0.0360 (15)	0.0115 (13)	0.0022 (12)	−0.0083 (13)
C6	0.064 (2)	0.0578 (19)	0.0297 (15)	0.0184 (16)	0.0014 (14)	0.0023 (13)
C7	0.0354 (13)	0.0425 (14)	0.0271 (12)	−0.0005 (11)	0.0007 (10)	−0.0099 (11)
C8	0.0405 (14)	0.0440 (15)	0.0341 (14)	−0.0037 (11)	0.0065 (11)	−0.0139 (12)
C9	0.061 (2)	0.088 (3)	0.065 (2)	−0.010 (2)	0.0353 (19)	−0.024 (2)
C10	0.0394 (17)	0.088 (3)	0.072 (2)	0.0077 (16)	−0.0050 (16)	−0.039 (2)
Cu1	0.02545 (18)	0.03205 (19)	0.02278 (18)	−0.00127 (12)	0.00141 (12)	−0.00789 (13)
N1	0.0349 (11)	0.0380 (11)	0.0270 (11)	−0.0005 (9)	0.0026 (9)	−0.0096 (9)
N2	0.0591 (16)	0.0529 (15)	0.0328 (13)	0.0057 (12)	0.0109 (12)	−0.0028 (11)
N3	0.0402 (14)	0.0682 (18)	0.0511 (16)	0.0005 (12)	0.0134 (12)	−0.0163 (14)
O1	0.0521 (12)	0.0447 (11)	0.0411 (11)	−0.0074 (9)	−0.0005 (9)	−0.0228 (9)
O2	0.0323 (9)	0.0473 (11)	0.0312 (10)	0.0073 (8)	0.0004 (7)	−0.0048 (8)
O3	0.0438 (10)	0.0409 (10)	0.0390 (10)	−0.0095 (8)	−0.0003 (8)	−0.0169 (8)
O4	0.0307 (9)	0.0441 (10)	0.0313 (9)	0.0039 (7)	−0.0032 (7)	−0.0070 (8)

### Geometric parameters (Å, °)

**Table:**

C1—O2^i^	1.252 (3)	C7—H7	0.9300
C1—O4	1.258 (3)	C8—N2	1.342 (4)
C1—C2	1.507 (3)	C8—N3	1.358 (4)
C2—H2A	0.9600	C9—N3	1.451 (4)
C2—H2B	0.9600	C9—H9A	0.9600
C2—H2C	0.9600	C9—H9B	0.9600
C3—O3	1.255 (3)	C9—H9C	0.9600
C3—O1^i^	1.259 (3)	C10—N3	1.447 (5)
C3—C4	1.506 (4)	C10—H10A	0.9600
C4—H4A	0.9600	C10—H10B	0.9600
C4—H4B	0.9600	C10—H10C	0.9600
C4—H4C	0.9600	Cu1—O3	1.9649 (18)
C5—N1	1.331 (4)	Cu1—O1	1.9654 (19)
C5—C6	1.363 (4)	Cu1—O2	1.9738 (18)
C5—H5	0.9300	Cu1—O4	1.9756 (18)
C6—N2	1.331 (4)	Cu1—N1	2.197 (2)
C6—H6	0.9300	Cu1—Cu1^i^	2.6326 (6)
C7—N1	1.321 (3)	O1—C3^i^	1.259 (3)
C7—C8	1.411 (4)	O2—C1^i^	1.252 (3)
			
O2^i^—C1—O4	125.7 (2)	H9A—C9—H9C	109.5
O2^i^—C1—C2	116.0 (2)	H9B—C9—H9C	109.5
O4—C1—C2	118.3 (2)	N3—C10—H10A	109.5
C1—C2—H2A	109.5	N3—C10—H10B	109.5
C1—C2—H2B	109.5	H10A—C10—H10B	109.5
H2A—C2—H2B	109.5	N3—C10—H10C	109.5
C1—C2—H2C	109.5	H10A—C10—H10C	109.5
H2A—C2—H2C	109.5	H10B—C10—H10C	109.5
H2B—C2—H2C	109.5	O3—Cu1—O1	168.16 (8)
O3—C3—O1^i^	125.6 (2)	O3—Cu1—O2	89.93 (9)
O3—C3—C4	117.4 (3)	O1—Cu1—O2	90.44 (9)
O1^i^—C3—C4	117.0 (2)	O3—Cu1—O4	88.93 (8)
C3—C4—H4A	109.5	O1—Cu1—O4	88.32 (9)
C3—C4—H4B	109.5	O2—Cu1—O4	168.35 (7)
H4A—C4—H4B	109.5	O3—Cu1—N1	94.74 (8)
C3—C4—H4C	109.5	O1—Cu1—N1	97.07 (8)
H4A—C4—H4C	109.5	O2—Cu1—N1	92.23 (8)
H4B—C4—H4C	109.5	O4—Cu1—N1	99.42 (8)
N1—C5—C6	120.7 (3)	O3—Cu1—Cu1^i^	83.66 (6)
N1—C5—H5	119.7	O1—Cu1—Cu1^i^	84.71 (6)
C6—C5—H5	119.7	O2—Cu1—Cu1^i^	81.05 (6)
N2—C6—C5	123.5 (3)	O4—Cu1—Cu1^i^	87.31 (5)
N2—C6—H6	118.3	N1—Cu1—Cu1^i^	173.08 (6)
C5—C6—H6	118.3	C7—N1—C5	117.8 (2)
N1—C7—C8	121.3 (3)	C7—N1—Cu1	121.20 (18)
N1—C7—H7	119.3	C5—N1—Cu1	120.88 (18)
C8—C7—H7	119.3	C6—N2—C8	116.2 (2)
N2—C8—N3	117.6 (3)	C8—N3—C10	121.5 (3)
N2—C8—C7	120.4 (3)	C8—N3—C9	120.0 (3)
N3—C8—C7	122.0 (3)	C10—N3—C9	118.4 (3)
N3—C9—H9A	109.5	C3^i^—O1—Cu1	122.29 (17)
N3—C9—H9B	109.5	C1^i^—O2—Cu1	126.74 (17)
H9A—C9—H9B	109.5	C3—O3—Cu1	123.65 (17)
N3—C9—H9C	109.5	C1—O4—Cu1	119.15 (16)
			
N1—C5—C6—N2	1.7 (6)	O3—Cu1—O1—C3^i^	−13.5 (5)
N1—C7—C8—N2	0.9 (4)	O2—Cu1—O1—C3^i^	78.2 (2)
N1—C7—C8—N3	−178.1 (3)	O4—Cu1—O1—C3^i^	−90.2 (2)
C8—C7—N1—C5	0.3 (4)	N1—Cu1—O1—C3^i^	170.5 (2)
C8—C7—N1—Cu1	−176.6 (2)	Cu1^i^—Cu1—O1—C3^i^	−2.7 (2)
C6—C5—N1—C7	−1.5 (5)	O3—Cu1—O2—C1^i^	81.6 (2)
C6—C5—N1—Cu1	175.3 (3)	O1—Cu1—O2—C1^i^	−86.6 (2)
O3—Cu1—N1—C7	85.5 (2)	O4—Cu1—O2—C1^i^	−2.8 (5)
O1—Cu1—N1—C7	−95.3 (2)	N1—Cu1—O2—C1^i^	176.3 (2)
O2—Cu1—N1—C7	−4.6 (2)	Cu1^i^—Cu1—O2—C1^i^	−2.0 (2)
O4—Cu1—N1—C7	175.2 (2)	O1^i^—C3—O3—Cu1	−1.4 (4)
Cu1^i^—Cu1—N1—C7	9.2 (6)	C4—C3—O3—Cu1	178.70 (18)
O3—Cu1—N1—C5	−91.3 (2)	O1—Cu1—O3—C3	13.4 (5)
O1—Cu1—N1—C5	87.9 (2)	O2—Cu1—O3—C3	−78.4 (2)
O2—Cu1—N1—C5	178.6 (2)	O4—Cu1—O3—C3	90.0 (2)
O4—Cu1—N1—C5	−1.6 (2)	N1—Cu1—O3—C3	−170.7 (2)
Cu1^i^—Cu1—N1—C5	−167.6 (4)	Cu1^i^—Cu1—O3—C3	2.6 (2)
C5—C6—N2—C8	−0.4 (5)	O2^i^—C1—O4—Cu1	3.5 (4)
N3—C8—N2—C6	178.2 (3)	C2—C1—O4—Cu1	−176.12 (18)
C7—C8—N2—C6	−0.8 (5)	O3—Cu1—O4—C1	−85.17 (19)
N2—C8—N3—C10	178.6 (3)	O1—Cu1—O4—C1	83.31 (19)
C7—C8—N3—C10	−2.4 (5)	O2—Cu1—O4—C1	−0.7 (5)
N2—C8—N3—C9	2.7 (5)	N1—Cu1—O4—C1	−179.80 (18)
C7—C8—N3—C9	−178.2 (3)	Cu1^i^—Cu1—O4—C1	−1.47 (18)

Symmetry codes: (i) −*x*+1, −*y*+1, −*z*+2.
